# Dopaminergic and Noradrenergic Contributions to Functionality in ADHD: The Role of Methylphenidate

**DOI:** 10.2174/157015908787386069

**Published:** 2008-12

**Authors:** Veronika Engert, Jens C Pruessner

**Affiliations:** Douglas Mental Health University Institute, Department of Psychiatry, McGill University, Montreal, Quebec, Canada H4H 1R3

**Keywords:** ADHD, methylphenidate, dopamine, noradrenaline, subcortical, prefrontal.

## Abstract

Attention Deficit Hyperactivity Disorder (ADHD) is a childhood psychiatric condition characterized by severe impulsiveness, inattention and overactivity. Methylphenidate (MPH), a psychostimulant affecting both the dopaminergic and the noradrenergic systems, is one of the most frequently prescribed treatments for ADHD. Despite the widespread use of MPH and its proven effectiveness, its precise neurochemical mechanisms of action are under debate. For the most part, MPH’s influence on subcortical dopamine neurotransmission is thought to play a crucial role in its behavioral and cognitive effects. In their hypothesis of biphasic MPH action, Seeman and Madras [42, 43] suggest that therapeutic doses of MPH elevate tonic dopamine while inhibiting phasic transmitter release in subcortical structures, leading to reduced postsynaptic receptor stimulation and psychomotor activation in response to salient stimuli. Volkow and colleagues [56] suggest that by amplifying a weak striatal dopamine signal, MPH increases the perception of a stimulus or task as salient. The enhanced interest for the task is thought to increase attention and improve performance. Recent animal studies have however shown that when administered at doses producing clinically relevant drug plasma levels and enhancing cognitive function, MPH preferentially activates dopamine and noradrenaline efflux within the prefrontal cortex relative to the subcortical structures [5]. Overall, we suggest that the delineated theories of MPH therapeutic action should not be discussed as exclusive. Studies are outlined that allow integrating the different findings and models.

## INTRODUCTION

Attention Deficit Hyperactivity Disorder (ADHD) is a childhood psychiatric condition characterized by severe impulsiveness, inattention and overactivity, often resulting in long-term educational and social disadvantage [50]. There is an ongoing debate concerning the core dysfunctions in ADHD, with the main stream of neuropsychological research putting emphasis on deficits in arousal (early stage information processing), activation (response preparation and readiness to respond), response inhibition and reward responding (for a review see [37]). 

The neural pathways underlying these deficits point mostly to frontal-subcortical catecholamine networks. More precisely, the nigrostriatal dopamine pathway projecting from the substantia nigra to the basal ganglia (caudate nucleus and putamen) is involved in motor control [47]. The mesolimbic dopamine pathway, projecting from the ventral tegmental area (VTA) to subcortical limbic regions (e.g. nucleus accumbens, olfactory tubercle and amygdala) is involved in motivated behavior and reinforcement learning (for a review see [24,  41]). The mesocortical dopamine pathway, also originating in the VTA, and projecting to the prefrontal cortex, is involved in cognitive functioning [17]. Eventually, noradrenergic projections ascending from the locus coeruleus to subcortical and cortical structures are involved in arousal and cognitive functioning [4,  6,  7].

Methylphenidate (MPH), a psychostimulant affecting both the dopaminergic and the noradrenergic systems, is one of the most frequently prescribed treatments for ADHD. In the 1930s, Charles Bradley first used a psychostimulant (the amphetamine sulfate benzedrine) as part of a workup of normally healthy children with neurological and behavioral problems. Although Bradely’s intention was to treat post-lumbar puncture headaches, he was surprised to find a dramatic improvement in the children’s learning and behavior. However, given that the use of medication in children was extremely controversial at that time, the discovery of benzedrine was largely ignored until MPH was found to be effective in the treatment of children with attention disorders in the 1960s (for a review see [46]). In ADHD patients, MPH reduces symptoms of impulsiveness, overactivity and inattention in up to 70% of affected children [23,  49]. Typical positive performance effects in healthy adults following single oral MPH doses include the improvement of vigilance, reaction time and working memory [8,  11,  13,  36]. Although the drug has been used for decades, the neural mechanisms underlying its – initially contraintuitive – clinical actions are still unknown.

Aim of the present review is to outline the most discussed catecholaminergic theories on the functionality of MPH in ADHD, and to present some preliminary data on MPH function in healthy volunteers.

## NEUROCHEMICAL MECHANISMS OF MPH

Despite the widespread use of MPH today – in Canada MPH prevalence in children aged 2 to 11 years ranged from 0.09% to 3.89% between 1994 and 1995 [40] – its precise neurochemical mechanisms of action are under debate. For the most part, MPH’s influence on dopamine neurotransmission is thought to play a crucial role in its behavioral and cognitive effects. The indirect dopamine agonist binds to the dopamine transporter (DAT). The DAT is the main mechanism by which the dopamine terminal removes dopamine released in response to a salient stimulus. By regulating the concentration of dopamine in the synapse, the DAT determines both the magnitude and the duration of the dopaminergic signal. Therefore, MPH-induced blockade of the DAT increases dopamine concentrations in the synapse and extracellular space [56]. Given an estimated median effective dose of 0.25mg/kg for oral MPH, therapeutic drug doses (0.3-0.6mg/kg) can be expected to occupy more than 50% of the DAT [57]. Highest specific MPH binding was found in terminal regions of the nigrostriatal and mesolimbic pathways (caudate-putamen, nucleus accumbens, olfactory tubercle and bed nucleus of the stria terminalis) [52]. These dopamine-rich subcortical structures have accordingly been hypothesized to mediate the drug’s clinical actions [42,  43,  56]. However, next to its dopamine-specific influence, MPH increases extracellular levels of the neurotransmitter noradrenaline by blocking its reuptake [18,  29]. Using microdialysis in rats, Berridge and colleagues [5] showed that low-dose MPH activates catecholamine neurotransmission within prefrontal structures. When administered at doses producing clinically relevant drug plasma levels and enhancing cognitive function, MPH preferentially activated dopamine and noradrenaline efflux within the prefrontal cortex relative to the subcortical nucleus accumbens. Since at the present time no radioligands are known to image the noradrenaline transporter, and imaging procedures are unable to reliably visualize the low levels of dopamine and noradrenaline actions in the cortex, the relevance of MPH’s noradrenergic and cortical effects has however not been investigated using functional imaging.

Next to effects on activity and self-regulation, increases in heart rate and blood pressure are characteristic side effects of MPH after single oral and intravenous drug doses as well as after long-time treatment [38,  51,  58]. Although these cardiovascular drug effects have been linked mainly to the noradrenergic system, changes in striatal dopamine seem to be crucially involved [58]. Moreover, MPH affects mood and arousal. Generally, the subjective drug effects are believed to be more reliably provoked by large and fast dopamine increases (as after insufflation or intravenous drug administration) [55], but nevertheless have been shown to occur after administration of oral doses [9].

## THEORIES ON THE FUNCTIONALITY OF MPH IN ADHD

Two hypotheses have been proposed to explain the clinical relevance of subcortical DAT blockade by MPH. The first hypothesis suggests that subsequent to DAT blockade, increased extracellular dopamine activates only the very sensitive presynaptic dopamine autoreceptors, which will lead to an attenuation of dopamine release in response to a salient stimulus [42,  43]. The second hypothesis suggests that increased extracellular dopamine subsequent to DAT blockade overcomes the inhibitory effects for activation of the presynaptic autoreceptors, leading to a net effect of dopamine accumulation in the synapse and subsequent amplification of dopamine signals [56]. Eventually, a third hypothesis shifts the focus from subcortical dopaminergic to prefrontal noradrenergic mechanisms in explaining MPH’s clinical actions [2,  5].

### Hypothesis of Biphasic MPH Action

Seeman and Madras [42,  43] suggest that the therapeutical relevance of subcortical DAT blockade by MPH is based on the drug’s biphasic action. Their hypothesis of biphasic MPH action, which builds on Grace’s tonic/phasic model of dopamine system regulation, aims to explain why psychomotor activity is reduced by low doses, and increased by high doses of psychostimulants.

According to Grace [19,  20], phasic release refers to the transient release of dopamine, produced by action potentials of dopamine neurons in response to behaviorally relevant external stimuli. Phasic release is competent to set free dopamine levels in the μM range [35]. This large but brief pulse of dopamine into the synaptic cleft is suggested to activate postsynaptic dopamine receptors and evoke dopamine-dependent behavioral responses [16,  22]. Before phasic dopamine diffuses into the extrasynaptic space, it is removed from the synaptic cleft by high capacity re-uptake systems within seconds. Unlike spike-dependent neurotransmitter increases reached within the synaptic cleft, dopamine levels in extrasynaptic fluid range between only 10-50nM [10,  44]. This extrasynaptic neurotransmitter concentration is under strong homeostatic control, as it is maintained even after the 6-hydroxydopamine (OHDA)-induced depletion of up to 80% of striatal dopamine [1,  39]. Despite present in low concentration, extrasynaptic dopamine seems to cause a steady-state partial activation of D2-like dopamine autoreceptors, which are located on dopamine neuron terminals. Any changes in extrasynaptic neurotransmitter levels are detected and counterregulated by these sensitive autoreceptors. Because of its tight control and slow time course of change, this phenomenon has been labeled tonic dopamine regulation [19]. Tonic dopamine release is regulated via presynaptic N-methyl-d-aspartate (NMDA) receptors in a spike-independent manner by glutamatergic prefrontal cortical afferents. As illustrated above, the presence of tonic dopamine in the extrasynaptic space provides a background stimulation of the sensitive presynaptic dopamine autoreceptors. Abnormal activation of these autoreceptors will trigger inhibition of neurotransmitter synthesis [25] and attenuation of phasic release [15].

Seeman and Madras [42,  43] argue that the basal or resting level of extracellular subcortical dopamine is approximately 4nM, and transiently rises at least 60-fold to about 250nM during a normal nerve impulse. This elevated level of extracellular dopamine falls back to 4nM within milliseconds, primarily by diffusion, but assisted by the dopamine transporter. When the DAT is blocked in the presence of a low therapeutic MPH dose, the resting level of extracellular dopamine rises by about 6-fold. This elevated resting state dopamine level is hypothesized to act on the presynaptic dopamine D2-like autoreceptors and consequently reduce the relative rise in impulse-triggered dopamine release to only twofold. A relative smaller pulsatile dopamine surge in response to a salient stimulus would therefore result in less activation of postsynaptic dopamine D1 and D2-like receptors, eventually resulting in reduced psychomotor activity in response to the stimulus. In accordance with this hypothesis, low doses of MPH have been shown to suppress locomotor activity in the rat under conditions associated with elevated arousal [31], similar to what is observed in ADHD. At higher MPH doses, the magnitude of the increase is argued to markedly raise both the resting level of extracellular dopamine and the pulsatile dopamine output, thereby causing widespread stimulation of postsynaptic dopamine receptors, overcoming the presynaptic inhibition of further neurotransmitter release, and triggering generalized stimulation of the nervous system (for a review see [42,  43]). 

### “Saliency Enhancing” Model

Volkow and colleagues [56] suggest that the therapeutical relevance of subcortical DAT blockade by MPH is based on two mechanisms. For one thing, dopamine is known to decrease background firing of striatal neurons while strengthening corticostriatal signals. Thereby, amplification of extracellular dopamine levels increases the signal-to-noise ratio in striatal target neurons [26]. In individuals with ADHD, the MPH-induced amplification of the striatal dopamine signal could thus improve attention and reduce distractibility. For another thing, mesolimbic dopamine signals the saliency of stimuli and drives motivation to perform goal-directed behavior (for a review see [24,  41]). Volkow *et al*. [56] hypothesize that in individuals with ADHD, the amplification of the dopamine signal could cause an increased perception of a stimulus as salient, thus motivating the individual to engage in a specific task, and improving attention and performance.

To test their hypothesis, Volkow and colleagues used Positron Emission Tomography (PET) and the dopamine D2-like receptor radiotracer [^11^C]raclopride. [^11^C]raclopride competes with endogenous dopamine for access to subcortical (predominantly basal ganglia) dopamine D2-like receptors. PET studies using this tracer provide an indirect measure of changes in synaptic dopamine levels. In a first study [54], ten healthy participants were administered either a 20mg dose of oral MPH or placebo. They were then presented appetitive food stimuli (visual and olfactory presentation of food items) and neutral stimuli (description of family genealogy). Participants additionally gave self-reports for their „desire for the food“ and „hunger“. MPH significantly increased dorsal striatal dopamine when given with salient food stimuli, but not when given with neutral stimuli. No such differences between the two types of stimuli were found after placebo administration. Stimulus presentation alone was therefore not strong enough to significantly increase striatal dopamine. MPH as compared to placebo also increased ratings of “desire for the food” and “hunger”, and these increases were correlated with dorsal striatal dopamine levels. In a second study [59], sixteen healthy participants were again administered either a 20mg dose of oral MPH or placebo. They then performed either an academic task (mathematical problems with monetary reinforcement) or a neutral task (passive viewing of nature pictures without remuneration). Participants additionally gave self-reports of how “exiting”, “interesting” and “motivating” they found the task. MPH significantly increased striatal dopamine only when given with the academic task. Again, no such differences between the two types of tasks were found after placebo administration. MPH as compared to placebo again increased rating of the task as “exiting”, “interesting” and “motivating”, and these increases were correlated with striatal dopamine levels. These findings support the hypothesis that by amplifying the dopamine signal, MPH increases the perception of a stimulus or task as salient. The enhanced interest for the task could increase attention and improve performance [56].

### Contribution of Prefrontal Noradrenaline

Much of the ADHD field has focused on subcortical dopaminergic mechanisms in explaining the therapeutic relevance of MPH. However, there is general agreement that ADHD involves weakened prefrontal cortex (PFC) function [3]. Deficits in PFC function are typically associated with hyperactivity, poor impulse control, distractibility, forgetfulness and poor organization/planning [48], all of which constitute symptoms of ADHD. Imaging studies have shown that MPH produces more efficient PFC function in ADHD patients and healthy controls [36,  53]. While both dopamine and noradrenaline have critical influence on PFC cognitive functioning, there are relatively low DAT levels in the PFC [7], a fact accentuating the potential importance of the neurotransmitter noradrenaline in MPH’s therapeutic actions. Indeed, recent biochemical studies using low doses of MPH showed more potent effects of the drug on hippocampal noradrenaline than on striatal dopamine [30], while increasing both noradrenaline and dopamine release in the PFC [5]. Indirect support for MPH’s noradrenergic actions also comes from the finding that ADHD symptoms can be recreated by blocking α2 adrenoceptors in the monkey PFC using yohimbine infusions [33,  34].

To examine the effects of low, clinically relevant MPH doses on PFC function in rats, Arnsten and Dudley [2] tested the animals on a spatial delayed alteration task, a classical working memory task of PFC function in rodents. To examine whether dopamine D1-like receptors and/or noradrenaline α2 adrenoceptor actions contributed to the effects of MPH on PFC function, either a D1-like or an α2 antagonist was co-administered. Results revealed that therapeutically relevant doses of MPH improved delayed alternation performance, and that this improvement likely reflected enhanced PFC cognitive function, since there were no changes in response time characteristics of motor or motivational changes. Both the D1-like and the α2 antagonists reversed the cognitive-enhancing effects of MPH. Although it cannot be said with certainty that the cognitive-enhancing MPH effects actually did occur in the PFC, Berridge *et al*. [5] provide additional animal data supporting this hypothesis. Using in vivo microdialysis in rats, the authors examined the degree to which clinically relevant MPH doses influenced dopamine and noradrenaline neurotransmission within the PFC as opposed to the subcortical nucleus accumbens. Again, the administered MPH doses improved sustained attention and working memory while having minimal effects on locomotion and arousal. MPH dose-dependently increased dopamine and noradrenaline efflux in the nucleus accumbens and PFC. However, MPH had substantially smaller effects on dopamine efflux in the nucleus accumbens, and it produced substantially larger noradrenaline than dopamine efflux in the PFC.

The current data are consistent with the hypothesis that at clinical doses, MPH improves performance by increasing the availability of dopamine and noradrenaline, which in turn stimulate D1-like and α2 receptors, preferentially within the PFC. Importantly, these data indicate that the noradrenergic prefrontal actions of MPH are just as important as the drug’s dopaminergic effects.

## ASSESSING THE COMBINED IMPACT OF MPH AND REWARD IN HEALTHY CONTROL PARTICIPANTS

The activity-reducing property of low-dose MPH is well established in the treatment of hyperactive children. Low-dose MPH also suppresses locomotor activity in the rat under conditions associated with elevated arousal [31]. Aim of the following study was to examine whether MPH would accordingly unfold its activity-reducing property in behaviorally aroused healthy participants [14]. For this purpose, 41 male university students accomplished a card-sorting task using monetary reward in a double-blind, placebo-controlled, between-group study design. We examined how behavioral activity in the card-sorting task was influenced (a) by monetary reward, which we expected to initiate phasic dopamine release in striatal structures [27,  28], (b) by a low therapeutic (20mg) dose of oral MPH, which we expected to enhance tonic dopamine availability and (c) by the combination of the two “stimulants”. We hypothesized to find an increase in behavioral activity in the reward as compared to the non-reward condition. According to Seeman and Madras’ hypothesis of biphasic MPH action, this reward-induced increase in behavioral activity should be lower after MPH as compared to placebo administration.

Aiming to acquire simple output measures of behavioral activity and performance accuracy, we assessed the numbers of total, correct and incorrect responses, and the success rate achieved in the task. Behavioral activity was represented by the number of total responses (composed of the number of correct and incorrect responses). Performance accuracy was represented by the number of errors and the success rate (defined as the ratio of correct to total responses). The card-sorting task comprised a non-reward and a reward condition. During the reward condition participants received 0.10$ for every correct response. The total reward could amount up to 12$. 

**Fig. (1):**
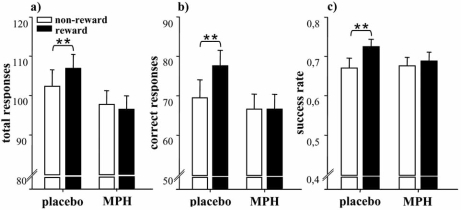
Means and standard errors for the interaction effect of reward and treatment on performance. Post-hoc simple main effects analyses revealed that after placebo as compared to methylphenidate (MPH) administration, a) the number of total responses (p=.02), b) the number of correct responses (p<.001) and c) the success rate (p<.001) were increased with reward. MPH thus inhibited a reward-induced increase in behavioral activity and performance improvement **p<.01.

Data analysis revealed that reward alone improved performance accuracy in the utilized card-sorting task by increasing the participants’ success rate (increasing the number of correct responses and decreasing the number of incorrect responses). MPH inhibited a reward-induced increase in both behavioral activity and performance accuracy by decreasing the achieved number of total and correct responses and consequently decreasing the success rate to the non-reward level. MPH thus equalized non-rewarded and rewarded performance (see Fig. **1**).

To the best of our knowledge, this is the first study to demonstrate MPH’s activity-reducing action in healthy participants. Although dopamine levels were not directly measured, the results support the notion that MPH produces biphasic effects via modulation of tonic and phasic dopamine. In accordance with Seeman and Madras’ hypothesis of biphasic MPH action [42,  43], heightened striatal tonic dopamine levels following drug challenge might have acted primarily on presynaptic dopamine autoreceptors. In turn, these autoreceptors might have initiated the relative reduction of stimulus-induced pulsatile transmitter release. A reward-induced increase in response-activity – determining the number of total and correct responses – might have consequently been inhibited. It is a limitation of this study that only one dose of MPH was tested. To ensure our conclusion of biphasic MPH action, a low and a high drug dose should have been tested against each other.

This study was not designed to test Volkow and colleague’s “saliency enhancing” model in parallel. It would have been necessary to measure the influence of MPH on the participants’ rating of task saliency as well as their motivation to engage in the task. According to Volkow *et al*., these measures should have been linked to attention and performance. Thus, rather than finding an attenuation in the number of correct responses, an increase in the number of correct responses and in success rate would have been expected with reward and MPH. Future investigations should be designed such that Volkow *et al*.’s “saliency enhancing” model [56] and Seeman and Madras’ hypothesis of biphasic MPH action [42,  43] can be tested against each other.

## CONCLUSION

Despite the widespread use of MPH in the treatment of ADHD, the neural mechanisms underlying the drug’s clinical actions are under debate. In this review, we summarized the three most discussed theories involving the catecholaminergic system.

Typically, each of these theories is discussed as exclusive. However, especially the neurochemical mechanisms suggested by Volkow and colleagues [56] and Seeman and Madras [42,  43] may be complimentary. Volkow *et al*. suggest that MPH amplifies stimuli-induced dopamine increases in magnitude and duration. Seeman and Madras, on the other hand, argue that the pulsatile dopamine surge elicited by a salient stimulus is decreased following the administration of a therapeutic MPH dose. Yet, the pulsatile dopamine surge is thought to be only *relatively* lower than it would have been in the absence of the drug (approximately threefold lower). Given the four possible states of extracellular dopamine levels a) at rest, b) during a nerve impulse, c) after MPH stimulation, and d) during a nerve impulse after MPH stimulation, the total amount of extracellular dopamine measurable in the striatum should be highest when a stimulus-triggered neurotransmitter release succeeds MPH challenge (approximately twofold higher than in the absence of the drug) [43]. Thus, the imaging results as found by Volkow *et al*. [54,  59] – an increase in striatal dopamine only if the dopamine-releasing effect of MPH is combined with either a salient food stimulus or task – would likewise be predicted by Seeman and Madras’ hypothesis. 

The main difficulty in integrating findings of subcortical and prefrontal MPH effects lies in the lacking comparability of available research methods. Whereas our knowledge of subcortical MPH effects stems mainly from imaging studies in humans using PET and the dopamine D2-like receptor radiotracer [^11^C]raclopride, MPH’s prefrontal actions are examined in the animal model using microdialysis. It can however be assumed that both the subcortical and prefrontal actions of MPH are implicated in its therapeutical action. Several researchers have effectively suggested basal ganglia and prefrontal cortex to be differentially involved in the motor and cognitive symptoms of ADHD (for a review see [12,  21,  45]), thus explaining differences in the specific efficacy of current dopaminergic and noradrenergic medication (for a review see [32]). On the basis of his tonic/phasic model of dopamine system regulation, Grace [21] thus argues that reduced stimulation from the prefrontal cortex determines low tonic dopamine activity in subcortical regions. Low tonic stimulation of inhibitory autoreceptors may in turn trigger increased phasic activity, which may again result in dysregulated motor and impulse control in ADHD patients. Aiming at the validation of this hypothesis, it would be of interest to repeat our test paradigm in a sample of ADHD patients with and without symptoms of hyperactivity, and to examine whether the achieved responsivity patterns dissociate between both types of the disorder.
